# Relationship between emotional divorce and alexithymia among married women in Saudi Arabia

**DOI:** 10.1186/s40359-023-01236-w

**Published:** 2023-08-02

**Authors:** Hend Faye AL-shahrani, Mohammad Ahmed Hammad

**Affiliations:** 1 Social Planning department, Faculty of Social Services, Princess Nourah bint Abdulrahman University, Riyadh city, Kingdom of Saudi Arabia; 2 Faculty of Education, Najran University, Najran city, Kingdom of Saudi Arabia; 3 Assiut University, El Fateh, Egypt

**Keywords:** Emotional divorce, Alexithymia, Woman, Saudi society

## Abstract

**Background:**

Emotional divorce occurs when a couple continues to live together due to necessity and coercion but they do not have a positive or constructive relationship, which negatively affects the stability of married life. Due to the low social acceptance of a formal divorce in the Saudi society, emotional divorce is common in several families. The rigidity of feelings and emotions within the family and the inability to express them may indicate the presence of alexithymia, which could result in the collapse of the family system and place the people involved at risk of mental health problems such as depression. Therefore, it is important to determine the prevalence of emotional divorce among married women in Saudi Arabia and to examine the relationship between emotional divorce and alexithymia.

**Methods:**

Data were collected from 305 married women in Saudi Arabia (M_age_ = 33.24 years; SD_age_ = 4.87 years), using the Emotional Divorce Scale and the Alexithymia Scale.

**Results:**

Results revealed that 78.36% of the participants experienced moderate to severe levels of emotional divorce. Working women, those who had been married for more than ten years, and those with five or more children exhibited a higher incidence of emotional divorce as compared to their counterparts. A linear regression analysis indicated that alexithymia was significantly associated with emotional divorce in this sample.

**Conclusion:**

These results suggest the need for examining the negative consequences of emotional divorce on the family and society. Additionally, it is important to educate young individuals of marriageable age about the nature of married life, and ways to deal with problems that occur. Finally, couples should be encouraged to express their positive and negative emotions with their spouse to build the marital relationship, and achieve compatibility and marital satisfaction.

**Supplementary Information:**

The online version contains supplementary material available at 10.1186/s40359-023-01236-w.

## Introduction

Divorce is a complex process. While it is not prohibited in Islam, Islamic law does not encourage Muslims to seek divorce. Marriage and the family remain very strong social institutions in Saudi Arabia, a country that strictly follows Islamic law, with Muslims forming a large majority of the population [1]. The formation and preservation of marriage are encouraged culturally and socially. Consequently, divorce continues to be largely discouraged in the Saudi culture, partly due to traditionalist views and partly due to the stigmatization of divorced couples, especially women [1, 2]. In addition to the limitations imposed by the cultural customs and traditions prevalent in the Saudi society, divorce is viewed as an economic burden as it carries particularly high costs for the family, women, and society [3]. As a result, the situation of couples who are unable to enjoy their marital relationship is getting worse. Furthermore, while such couples may not take the legal route to separation and obtain a formal divorce, their marital dissatisfaction may still lead to emotional divorce, or what is called “hidden divorce” [2]. Emotional divorce usually occurs when a couple withholds emotion and feelings from their spouse because they may not be able to express their feelings satisfactorily, or they are unable to comprehend and manage the intensity or contradiction of their feelings [4]. There is no single established definition of emotional divorce but it is commonly characterized by a lack of emotional understanding, marital dissatisfaction, lack of intimacy, apathy towards or disregard for each other’s’ feelings, and emotional distance [new references [5–7]. Researchers emphasize that emotional divorce may occur at any point in a marital relationship, including before or after legal separation and divorce, and irrespective of cohabitation status [8].

To avoid legal divorce owing to socio-cultural pressures, couples may continue to cohabitate and work together as a social team, but their mutual attraction and trust disappears and they become hostile to each other over time [9]. Specifically, in Arab countries, including Saudi Arabia, women who are dissatisfied with their marriage prefer not to divorce legally due to the socio-cultural pressures, and the economic and legal inequalities they face [10].

Though couples experiencing emotional divorce may continue to live together for social, cultural, legal, economic, and religious reasons [8, 11], couples and their children tend to experience several social, emotional, health, and psychological consequences such as depression, anxiety, depression, stress, aggressiveness, and psychometric disorders owing to the dysfunctional marital relationship [4, 8, 12]. Several previous studies have examined the causes of emotional divorce and its association with other variables. For instance, Ataya [13] reported that the loss of mutual understanding of the emotions and feelings of the other party, and the impaired ability to recognize and appreciate them are some of the most influential causes of emotional divorce. Oftentimes, a husband wishes for a wife who understands his emotions and satisfies his desires, while a wife dreams of someone who appreciates her feelings, understands them, and respects her privacy. In the absence of this mutual understanding of the partner’s emotions and lack of expression of one’s own feelings and needs the gap between spouses will widen, mutual discord will increase, and disagreements will deepen. A study by Hourani and Gharbawi [14] revealed the following as some common causes of emotional divorce: absence of a husband, lack of family communication, infidelity, absence of romantic/emotional touch, prolonged interruption of intimacy and emotional coldness.

Furthermore, other studies found that undermining one’s spouse in words or deeds, whether in front of other people or children, depression, arrogance or superiority of one partner over the other, and material pressures and cost of living were some leading causes of emotional divorce [9, 15, 16]. These studies also reported that emotional divorce had a negative effect on the woman’s self-esteem, which in turn negatively affects her interactions with family members. Khalifa [17] Asserted that owing to the social, technological, and economic changes occurring in contemporary societies, there has been a negative impact on spousal relationships, leading to the dismantling of bonds between family members, children’s suffering, and lack of gratitude between spouses. These changes, in turn lead to the occurrence of emotional divorce.

Similarly, a study by [18] found that the length of the marriage and the presence of a wife’s function may be some of the common causes of emotional divorce. Al-Najdawi [19] Found that differences in the spouses’ concept of marriage, which is formed through socialization and cultural influences such as male dominance, could lead to emotional divorce. Additionally, the pressure to maintain one’s social image, concern for children’s future, and fear of parents were identified as other reasons for not divorcing one’s spouse. Halchuk, Makinen [20] asserted that professionals could help couples enhance their ability to solve problems and better tackle marital challenges such as family insecurity; emotional apathy; and psychological, social, and behavioral problems within the family.

Evidently, several individual and personal variables play an important role in the phenomenon of emotional divorce. Alexithymia is one such individual factor, which includes characteristics such as the inability to express, describe, or distinguish between emotions [21]. It usually co-occurs with other psychological disorders and may be exacerbated by constant exposure to psychological stress [13]. It may also be a stand-alone disorder [22]. Alexithymia is a multidimensional concept that has four distinctive characteristics: (a) trouble with recognizing and describing emotions, (b) difficulty in distinguishing between emotions and physical stimuli, (c) imperfect fantasies, and (d) lower objective and introverted thinking (externally oriented) thinking) [23]. Numerous studies have shown that people with alexithymia experience difficulty in forming and maintaining emotional relationships, and in correctly recognizing the feelings of others [24]. Successful and satisfying marital relationships certainly require the ability to recognize, understand, and express feelings and emotions [23].

Emotional exchange and empathy are key factors in distinguishing between successful and unsuccessful marriages. It also plays an important role in the development of intimacy [25]. The mutual expression of feelings between couples also provides a sense of support and leads to greater self-disclosure, which in turn relates to marital compatibility [23, 26]. Studies have also indicated that alexithymia paves the way for problems in personal and marital life [23, 27]. People with alexithymia are more likely to use suppression strategies and less likely to use reassessment strategies as compared to those without this disorder [28]. Suppression strategies are often classified as negative coping strategies that are closely linked to physical and mental health problems [29]. A growing body of literature also suggests that alexithymia is a personality trait that significantly undermines relational quality among married couples [e.g., 30]. Alexithymia was found to be associated with lower levels of relationship satisfaction, sexual satisfaction, attachment security, and need for intimacy [30–32]. Additionally, alexithymia-linked depression has been associated with personal apathy, which reflects low expectations of others and a low desire to meet the expectations of others [33].

Hesse and Floyd [34] Conducted an observational study to examine the effect of alexithymia on primary reactions; Their findings revealed that participants reported being less physically and socially attracted to high alexithymia participants as compared to non-alexithymia participants, and they reported lower levels of intimate relational messages during a conversation with non-alexithymia participants. Relatedly, Frye-Cox and Hesse [35] examined the intermediate role of loneliness and intimate communication in the association between alexithymia and marital quality of life; Their results indicated that loneliness and intimate communication fully mediated the link between alexithymia and marital quality of life.

Although the impact of formal divorce has been well addressed by scholars both nationally and internationally [1, 3, 36–38], emotional divorce still needs more attention from researchers in Arab societies, especially in Saudi Arabia. Due to lack of data on the prevalence of emotional divorce in any context and the difficulty in identifying related variables, the present study aimed to examine the level of emotional divorce among married women in Saudi Arabia. Additionally, it attempted to examine if alexithymia could predict the incidence of emotional divorce.

## The current study

Emotional divorce is seen as one of the most common consequences of dissatisfaction among couples, leading to the emotional distress in one or both spouses [4]. Emotional divorce is undoubtedly frustrating and it is often accompanied by various traumas for couples, their children and possibly their extended families. In other words, emotional divorce is a process that begins with each spouse’s experience of emotional crisis and ends with an attempt to resolve the conflict by entering a new position with new roles and lifestyles [39]. Recently, the global divorce rate has risen rapidly due to social, economic, and cultural transformation [38]. This substantial increase has even been observed in traditionalist countries such as Saudi Arabia, that have significant social and cultural obstacles to divorce [1, 3]. According to statistics from Saudi Arabia, 37% of marriages end in divorce, which is substantially higher than the global average of 18–22%. Furthermore, most divorces occur during the first year of marriage, and up to 60% of those occur among newlyweds [40]. It would be interesting to examine the nature of socio-cultural changes that are causing these trends to change. However, that is not within the purview of the present study. Notably, despite high rates, these statistics only reflect the situation pertaining to formal divorce and it is unclear if the male or female partner initiates or pursues formal divorce. Considering the influences of patriarchy and religious laws that govern the Saudi society, women have weaker social and economic standing. Additionally, the legal rights of divorced women, especially pertaining to succession, property ownership, etc., are limited, again, partially owing to gender bias [9]. Therefore, Saudi women may be less likely to initiate formal divorce proceedings, are more likely to remain in a dysfunctional marriage. Furthermore, statistics on the incidence of emotional divorce between spouses are difficult to come by, with most studies suggesting that emotional divorce is twice as prevalent as formal divorce [15, 41].

Emotional divorce leads to increased mental health risks for individuals and families by significantly reducing their well-being [4, 14]. This multidimensional phenomenon can cause and exacerbate physical and psychological disorders such as neurological, cognitive, emotional, and behavioral diseases [42]. Alexithymia, which is described as a set of cognitive and emotional traits observed among patients with mental disorders [22], is therefore an influential variable that affects the couple and contributes to divorce. The role of alexithymia in the processing and modification of feelings has been studied extensively and it has been proven that it could pave the way for problems in personal and marital life [23, 27]. Since alexithymia can affect the regulation of emotions, its incidence could be closely associated with that of emotional divorce [43]. Although the effect of formal divorce on the involved individuals has been well addressed by scientists both nationally and internationally [3], emotional divorce and its prevalence, and its relationship to many psychological variables still needs more attention from researchers in traditionalist societies, especially in Saudi Arabia. As explained in the earlier in the paper, the Saudi social structure renders women more vulnerable to experiencing emotional divorce owing to social, cultural, religious, legal, and economic influences. Finally, it is well-established that emotional characteristics differ across genders. With differences in reasons for staying or not staying married, emotional skills, expectations from marriage, social roles, etc., among men and women, it was decided to limit the present study to female participants.

Given the research gaps identified in the preceding sections and the increasing pressure that the emotional divorce process places on Saudi women, the present study aimed to determine the incidence of emotional divorce among married women in Saudi Arabia, considering their demographic characteristics such as employment status, marriage duration, and number of children. These demographic characteristics were chosen because they may be impinging factors in Saudi women’s decision to stay married despite marital dissatisfaction. Further, the present study aimed to examine the relationship between emotional divorce and alexithymia. To achieve these objectives, the following research questions were explored:

1. What is the level of prevalence of emotional divorce among married women in Saudi Arabia?
2. Does the prevalence of emotional divorce vary based on other variables such as participants’ employment status, marriage duration, and number of children?
3. Is the level of alexithymia a predictor of emotional divorce?

## Methodology

### Sample

This descriptive cross-sectional study was conducted with married women in Saudi Arabia. Using G*Power version 3.1.9.7, we computed the recommended sample size for linear regression with 0.05 α error probability and 80% power. The resultant recommendation was 270 participants. Purposive sampling was used to select participants who fit the inclusion and exclusion criteria. The following selection criteria were utilized: volunteering to participate in the study, being aged over 18 years, being currently married, being able to read Arabic, being citizens of Saudi Arabia, not having any intellectual disabilities, and not having a formal diagnosis of a mental illness. The initial sample included 350 participants, of which, 10% later refused to participate. An additional 10 respondents were disqualified due to incomplete data. The final sample included 305 Saudi married women aged 21–55 years (M_age_ = 33.24 years; SD_age_ = 4.87 years), which was over the recommended sample size of 270 participants. Table 1 presents the detailed demographic information of the participants.

**Table 1:** Demographic characteristics of the study participants

Demographic Characteristics		N	Percentage
Employment status	Employed	189	61.96%
	Not employed	116	38.03%
Number of children	≤ 1 children	65	21.31%
	2–4 children	124	40.65%
	≥ 5 children	116	38.03
Marriage duration	≤ 4 years	89	29.18%
	5–9 years	94	30.81%
	≥ 10 years	122	40%

### Data collection

Data were collected in August 2022. Prior to data collection, potential participants were informed of the possibility of volunteering for our study via social media and were provided a link containing information about the research and the survey link. Participants were informed that their completion of the survey would be taken to indicate their consent for voluntary participation. It was also clarified that all information obtained would remain strictly confidential and that the data would only be used for research purposes. The survey link included a questionnaire on participants’ demographic information (See Appendix 1) and the two tools described below, namely, the Emotional Divorce Scale and the Toronto Alexithymia Scale-20.

Prior to its commencement, the study received ethical approval from the Deanship of Scientific Research at Princess Nourah Bint Abdul Rahman University (FRP-1443-25). In addition, all research procedures conformed to those outlined in the Helsinki Declaration. Data collection took two months.

#### The emotional divorce scale

The Arabic version of the 37-item Emotional Divorce Scale [44] was used. This tool assesses the level of emotional divorce in married couples. Items are presented across the following three dimensions: the social sphere (11 items), the psychological sphere (13 items), and the emotional sphere (13 items). Each item is rated on a 5-point Likert-type scale (1 = never, 2 = rarely, 3 = sometimes, 4 = high, and 5 = always), with a total possible score ranging from 37 to 185 points. The degree of emotional divorce is classified into three levels; high, medium, and low, using the following equation:

$$\begin{array}{c}\frac{{highestresponse - lowestresponse}}{{Numberofcategories}} = \frac{{5 - 1}}{3} = \\\frac{4}{3} = 1.33\end{array}$$

Accordingly, scale scores are classified as follows: < 2.33 as low emotional divorce, 2.34–3.67 as moderate emotional divorce, and > 3.68 as high emotional divorce.

The validity of the scale was confirmed by assessing its internal consistency based on the interrelationships between items [38]. Overall internal consistency scores ranged between 0.58 and 0.67. The Cronbach’s alpha for individual items ranged from 0.77 to 0.86, and that for the total scale score was 0.79, indicating optimal validity. The split-half coefficient was 0.89 [38].

#### The toronto alexithymia scale (TAS-20)

The TAS-20 was used as a measure of alexithymia [45]. This self-report 20-item scale contains three factors, namely, difficulties in recognizing feelings (DIF), difficulties in describing feelings (DDF), and externally oriented thinking (EOT). Each item is rated on a 5-point Likert-type scale (1 = strongly disagree, 5 = strongly agree). Total scores range from 0 to 100 with scores of 0 to 51 representing no alexithymia, 52 to 60 representing possible alexithymia, and 61–100 representing as presence of alexithymia. The original scale exhibited good psychometric properties in several studies [46–48]. The TAS-20 showed good convergent and characteristic validity, and the self-report scores exhibit high compatibility with observers’ ratings of alexithymia. Bagby, Taylor [45] found high internal consistency (r = 0.81) in their development studies. They also reported optimal test-retest reliability (r = 0.77) within three weeks. For use in the present study, the TAS-20 was translated from English to Arabic and then translated back into English by language experts, to ensure that the Arabic translation maintained the same meaning as the original questionnaire. Specifically, first, the English version was translated into Arabic by a bilingual professor from the Department of English at the researcher’s institution. The Arabic version was then retranslated into English by another professor who specialized in English and whose first language is Arabic. Subsequently, the translated Arabic and English versions were reviewed by three specialists in Arabic, Psychology, and English, respectively. Based on consensus among the three specialists, some words and items were revised to create the final Arabic version of the tool. No difficulties were found in using the tool in the Saudi context. In the present study, the Cronbach’s α for the Arabic version ranged from 0.76 to 0.83, indicating excellent reliability.

### Data analysis

All statistical analyses were conducted using SPSS version 20. Frequency and percentage were used to examine the prevalence of emotional divorce among Saudi married women. In addition, means, standard deviations, and t-tests were used for bivariate analyses considering demographic variables. Finally, linear regression analysis was used to examine the association between emotional divorce and alexithymia.

## Results

### Prevalence of emotional divorce

Table 2 shows the prevalence of emotional divorce among the present sample of Saudi married women, classified into low, moderate, and severe emotional divorce levels based on the aforementioned procedure. The frequency and distribution of married women experiencing different levels of emotional divorce were determined. A large majority of the participants (64.59%) exhibited moderate emotional divorce, followed by about a quarter of the sample (21.63%) who exhibited severe emotional divorce. The rest of the participants (13.77%) exhibited low emotional divorce.

**Table 2:** Prevalence of Emotional Divorce among Saudi Married Women

Emotional divorce scores	Emotional divorce level	Frequency (N = 305)	Percentage
≤ 2.33	Low emotional divorce	66	21.63%
2.34 to 3.67	Moderate emotional divorce	197	64.59%
≥ 3.68	Severe emotional divorce	42	13.77%

Table 3 presents the findings of the one-way ANOVA conducted to examine differences in the level of emotional divorce based on number of children, marriage duration, and employment status. With statistically significant findings at p < 0.01 for all variables, it is clear that Saudi married women’s emotional divorce levels differed based on these demographic characteristics.

**Table 3:** ANOVA results examining Emotional Divorce Scale Scores By Presence of Children, Marriage Duration, and Employment Status among Saudi Married Women

Source	Type III Sum of Squares	df	Mean Square	F	Sig.
Corrected Model	17799.507^a^	5	3559.901	18.825	0.000
Intercept	3552801.785	1	3552801.785	18787.809	0.000
Number of children	1662.132	2	831.066	4.395	0.013
Marriage duration	1872.344	2	936.172	4.951	0.008
Employment status	4758.819	1	4758.819	25.165	0.000
Error	56541.332	299	189.101		
Total	4395112.000	305			
Corrected Total	74340.839	304			

^a^R^2^ = 0.239 (Adjusted R^2^ = 0.227)

To further assess the nature of these differences, the Scheffe test was used for dimensional comparisons based on number of children (Table 4) and marriage duration (Table 5).

**Table 4:** Scheffe Test Comparison of Differences in Emotional Divorce based on Number of Children

(I) Number of children	(J) Number of children	Mean Difference (I-J)	Std. Error	Sig.	95% Confidence Interval	
					Lower Bound	Upper Bound
≤ 1 children	2–4 children	-15.24^*^	2.209	0.000	-20.68	-9.81
	≥ 5 children	-12.45^*^	1.812	0.000	-16.91	-7.99
2–4 children	≤ 1 children	15.24^*^	2.209	0.000	9.81	20.68
	≥ 5 children	2.79	2.055	0.399	-2.26	7.85
≥ 5 children	≤ 1 children	12.45^*^	1.812	0.000	7.99	16.91
	2–4 children	-2.79	2.055	0.399	-7.85	2.26

^*^ The mean difference is significant at the 0.05 level

**Table 5:** Scheffe Test Comparison of Differences in Emotional Divorce based on Marriage Duration

(I) Marriage duration	(J) Marriage duration	Mean Difference (I-J)	Std. Error	Sig.	95% Confidence Interval	
					Lower Bound	Upper Bound
≤ 4 years	5–9 years	-12.46^*^	2.034	0.000	-17.47	-7.46
	≥ 10 years	-10.76^*^	1.917	0.000	-15.48	-6.05
5–9 years	≤ 4 years	12.46^*^	2.034	0.000	7.46	17.47
	≥ 10 years	1.70	1.887	0.000	-2.94	6.34
≥ 10 years	≤ 4 years	10.76^*^	1.917	0.000	6.05	15.48
	5–9 years	-1.70	1.887	0.000	-6.34	2.94

^*^The mean difference is significant at the 0.05 level

Table 4 confirms the presence of statistically significant differences in the emotional divorce scores of Saudi married women based on the number of children they had. Specifically, emotional divorce scores of those with 1 or no child were significantly lower than scores of those with 2–4 or > 5 children, while there were no significant differences between those with 2–4 or > 5 children. Overall, these results suggests that women with 2 or more children were more likely to exhibit higher emotional divorce scores as compared to those with 1 or no child.

Table 5 shows statistically significant differences in emotional divorce based on marriage duration. Specifically, emotional divorce scores of those who had been married for 4 years or less were significantly lower than scores of those married for 5–9, or 10 or more years. However, there were no significant differences between those married for 5–9 years and 10 or more years. Overall, these results suggests that women who had been married relatively recently, i.e., for 4 years or less, had lower emotional divorce scores, while those married for 5 or more years were more likely to exhibit higher emotional divorce scores. That is, the longer the marriage lasts, the higher was the likelihood of experiencing emotional divorce in the study sample.

Table 6 presents findings of a t-test conducted to compare emotional divorce scores based on the participants’ employment status. Findings revealed that emotional divorce was more pronounced among working married women (M = 124.41, SD = 13.96) as compared to non-working married women (M = 111.65, SD = 14.61). Hence, the results suggest that not being employed may have had a mitigating effect on the level of emotional divorce experienced by the married women in the present sample.

**Table 6:** T-test Analysis of Emotional Divorce based on Employment Status

Employment status	N	M	SD	t
Employed	189	124.41	13.96	6.97^*^
Not employed	116	111.65	14.61	

^*^Statistically significant at p < 0.05 level

### Emotional divorce and alexithymia

The mean score on the TAD-20 was 65.9 (SD = 9.42, range = 34–84). Furthermore, a classification based on that recommended by the tool creators revealed that a substantial majority of the participants had alexithymia as per this screening tool (Table 7).

**Table 7:** *Classification of alexithymia scores based on recommended cutoffs*

TAS-20 classification	*f*	%
No alexithymia (0–51)	27	8.9
Possible alexithymia (52–60)	50	16.4
Alexithymia present (61–100)	228	74.8

^*^Statistically significant at p < 0.05 level

Combining the groups that possibly or definitely had alexithymia present showed that over 90% of the present sample showed several characteristics of alexithymia, which warranted further analyses to establish the association between emotional divorce and alexithymia. Table 8 presents the findings of the linear regression analysis examining the association between emotional divorce and alexithymia. As all three demographic variables were significantly correlated with the dependent variable, they were included as control variables in the analysis. Findings revealed that alexithymia was a significant predictor of emotional divorce. It explained 11.5% of the variance in emotional divorce scores (R = 0.339; R^2^ = 0.115; Adjusted R^2^ = 0.103; F_change_ = 6.18, p < 0.05). While it is a statistically significant predictor of emotional divorce scores, the direction of causality is unestablished, and evidently, major proportion of the variance remains unexplained by this model. As such, it is acknowledged that the rest of the variance can be attributed to other factors that need to be examined in future studies.

**Table 8:** Linear Regression Analysis of Association between Emotional Divorce and Alexithymia

Variables	Unstandardized coefficient		Standardized coefficient Beta	t	P
	B	Std. Error			
Alexithymia	0.137	0.036	0.227	3.758	0.00

Note. Employment status, marriage duration, and number of children were included as control variables

## Discussion

Continuing to cohabitate in the absence of feelings and emotions among spouses has several effects on different aspects of family life as a whole. The couple’s keenness to maintain their image in the society and the fear of declaring divorce leads them to change their lifestyle such that though they experience emotional divorce they keep up the façade of a happy married life. Such circumstances may only delay but not prevent eventual dissolution of the spousal relationship, while everyone in the family simultaneously experience the underlying tension and anxiety related to the dysfunctional relationship [2, 16]. Emotional divorce is a serious scourge that affects marital life. Further, it threatens the psychological integrity of the family and affects its stability and solidarity [18]. This phenomenon has become a tangible reality in the Saudi society for several reasons, with the most common ones being the couple’s inability to identify, distinguish, and express feelings; lack of understanding of physiological changes that accompany emotional arousal; and inability to engage in imaginary processing [18]. Together, these characteristics are called alexithymia [49]. With its increasing incidence and wide impacts, emotional divorce needs urgent attention from counselors and educators, especially since it affects not only the couple’s dyadic relationship but also their responsibilities and roles in the entire family system [2, 50].

Results of the present study revealed that 78.36% of the Saudi married women experienced moderate to severe levels of emotional divorce. This was evident from a range of behaviors assessed by the Emotional Divorce Scale, including the participants’ feeling of having to continue the marital relationship for the sake of their children, their husband’s lack of empathy for them when they are under strong pressure, their lack of connection or intimacy towards their husband, their dissatisfaction with marital life, non-existent sexual relationship between spouses, and their sense of distress when their husband approached them. Despite being remarkably high, the present prevalence rates were comparable to those reported in previous studies [6, 50–52].

Present findings also revealed that unemployed women experienced lower levels of emotional divorce as compared to working married women. It is well known that working women have to juggle the multiplicity of roles assigned to them, whether at home or at work [53, 54]. Consequently, some working women may tend to neglect their husbands and children owing to work pressures. This may lead their husband to lack interest in his wife owing to the absence of adequate time to exchange feelings with the other party. This dynamic may lead to severe psychological pressure for both the husband and wife. Furthermore, if the husband is ill equipped to support his working wife even on a psychological level, he may accuse her of negligence and irresponsibility. This, in turn may cause anxiety and guilt in the wife. Additionally, notwithstanding whether the wife shoulders exponentially more responsibilities than the husband, he may be convinced that her shortcomings on the family/marital front are attributable to her work. Thus, her work becomes a reason to blame all their differences upon. Sabban, Al-Samiri [18] reported similar findings from a study of 400 Saudi women working in different professions, including teachers, doctors, administrators, and teaching members. They found that only 40% of married women receive marital support, while 55% of working married women admitted that they do not receive support from their husbands. However, other studies have reported contradicting findings in that they observed higher levels of emotional divorce among unemployed women as compared to working women [2, 55, 56].

The current study also found that the duration of marriage affects the level of emotional divorce among Saudi married women. Specifically, it was observed that the older the marriage, the greater was the emotional divorce. This may be due to the fact that, with age, the wife may be bored of the routine of married life and this boredom leads to constant disagreements and decline in the language of love. There is no dialogue and no real communication, causing emotional divorce to occur. This may also be due to the many pressures and family problems that negatively affect the relationship of the couple, and this is indicated by the study conducted by [57], which showed a significant relationship between family pressures and emotional divorce. That study also confirmed that the level of emotional divorce can be predicted from the size of family pressures, as the neglection of couple’s crises and their failure to initiate problem-solving leads to distance between them. This, in turn, leads to the occurrence of emotional divorce. Additionally, it may lead one of the partners to abandon all their responsibilities and the other to shoulder all. Such a situation would cause both partners to withdraw from each other, ultimately leading to emotional divorce [58].

Another interesting finding of the present study was the effect of number of children on the level of emotional divorce. Families with two or more children were found to have higher levels of emotional divorce. This may be due to the fact that a larger family would impose higher burdens and responsibilities on the woman, causing her to be overwhelmed with household and child-rearing responsibilities as well as marital responsibilities. Furthermore, having a larger family would lead to higher burden on the husband to meet the needs of the family. As such, the couple may be likely to neglect their dyadic relationship, and have poor verbal and physical communication, thus leading to emotional divorce. This finding is consistent with those reported in previous studies [19, 59, 60]. Alternatively, it may be easier for couples with no or fewer children to obtain a legal divorce, especially considering custody issues. In contrast, those with more children may feel compelled to stay together despite emotional difficulties, as their responsibilities towards their children take precedence.

Finally, the current results suggested an association between emotional divorce and alexithymia, such that the latter explained 11.5% of the variance in emotional divorce when controlling for the effects of marriage duration, number of children, and employment status. This can be explained by the fact that the dimensions of alexithymia, namely, difficulties in recognizing feelings, difficulties in describing feelings, and externally directed thinking, can predict emotional divorce between spouses. Lack of emotional communication and empathy foreshadow emotional divorce [61]. Each partner’s awareness of their own emotions and their ability to perceive and express them appropriately and satisfactorily to their life partner are fundamental factors in both individuals’ sense of satisfaction with their partner, and with life in general. In addition, their emotional communication and regulation abilities contribute to their understanding of the verbal and non-verbal cues of their partner, and to their ability to solve conflicts and enjoy marital harmony. As individuals with a high level of alexithymia lack these abilities, it may lead to the failure of the marital relationship, and thus, the emergence of emotional divorce. The results of the present study are consistent with results of previous studies [21, 61]. They found that alexithymia leads the individual to receive less social support, and to experience dissatisfaction with the marital relationship and poor intimacy. As such, the individual may be more likely to experience depression. Similarly, Ataya [13] reported that alexithymia is one of the most important predictors of marital dissatisfaction, weak marital relationships, and emotional divorce. Other studies have also reported alexithymia to be negative correlated with marital satisfaction and interpersonal relationships. Specifically, within interpersonal spousal relationships, their sexual relationship and sexual satisfaction was found to be most important for achieving marital satisfaction [32, 49]. It is important to note that there may be a bidirectional relationship between emotional divorce and alexithymia, which cannot be explained using the present data and study design. Nevertheless, experiencing emotional divorce and the feeling of being stuck in an unhappy marriage may influence the sense of apathy and emotional distance that characterize alexithymia. These findings have important implications for practice, but before discussing them, it is essential to acknowledge the study limitations.

### Limitations of the study

The present sample was had inherent biases stemming from the sampling and data collection methods. Specifically, purposively sampling volunteers through and advertisement posted on social media rendered the sample biased towards those with access to social media, internet services, and possibly education too. These participants may not be representative of the broader population, as evident from a higher proportion of the present sample comprising working women. Furthermore, the sample is not divergent enough to reflect the social reality of Saudi Arabia, which includes women who do not meet one or more of the selection criteria set for this study.

Further, the sample size was quite small to be representative of the national population of married women. However, this sample size was estimated using the G*Power tool’s recommendations for preset power and error limits. Therefore, there is some statistical soundness that the sample of 305 women offers. Nevertheless, it is recommended that future studies include a larger, more diverse and representative sample to provide a clearer picture of the general population. Finally, in regard to the sample, the study also excluded male perspectives by including only female participants. Future comparative studies including males and females would provide a more holistic understanding of the core variables studied.

This study could have also benefitted from including more demographic variables in the bivariate analyses pertaining to the incidence of emotional divorce, and as potential control variables in the regression analysis. Regarding the association between the two study variables, though alexithymia was a significant predictor of emotional divorce, it only explained 11.5% of the variance in the present study. This could be, in part, be attributed to the present sample’s limitations. However, more importantly, this points to the fact that spousal relationships are influenced by several co-occurring and closely interconnected factors. Unfortunately, other factors could not be included in the present study. It is strongly recommended for future studies to simultaneously study factors such as individual factors (personality, identity factors, personal beliefs and expectations about married life, emotional maturity, presence or comorbid or pre-existing psychological problems, etc.), marital factors (nature of marriage: arranged/love, dyadic communication, family structure, etc.), social factors (gender roles, social privilege, access to and views on professional help, individualistic vs. collectivistic culture, etc.), and several others.

Finally, the cross-sectional design employed in the study did not allow for the establishment of causality and its direction with reference to emotional divorce and alexithymia. Indeed, both variables could have a bidirectional causal relationship among themselves and with other intervening variables. Future studies should attempt to deconstruct the causal mechanism in play.

## Conclusions and implications

The main objective of this study was to determine the prevalence of emotional divorce among married women in Saudi Arabia and to examine the relationship between emotional divorce and alexithymia. The results revealed that majority of the women experienced moderate to severe emotional divorce, with statistically significant differences based on participants’ employment status, marriage duration, and number of children. Specifically, the results showed that working married women, those who were married for longer, and those with a greater number of children tended to experience higher levels of emotional divorce as compared to their counterparts. In addition, the results of the linear regression analysis indicated that alexithymia predicted 6% of the variance in emotional divorce. The high proportion of the variance was still unexplained. This highlights the fact that other factors that influence the level of emotional divorce among married women in Saudi Arabia need to be identified.

Finally, though the present findings do not clarify the direction of the relationship between emotional divorce and alexithymia, present and previous research provides strong evidence of the negative impact of emotional divorce on the psychological well-being of couples and children. Therefore, it is important to recognize this as a public health concern and to develop appropriate strategies to control emotional divorce levels through establishing emotional skills.

Specifically, the need to educate adults of marriageable age about the nature of married life, ways of dealing with marital and family problems, and ways to strengthen the marital relationship through emotional expression is evident. Such pre-marital education could reduce the chances of emotional divorce or even alexithymia by helping young married couples to achieve compatibility, emotional stability, and marital satisfaction. Moreover, it is important to increase public awareness about the seriousness of emotional divorce and how to eliminate it in the society. Dissatisfaction with marriage is commonplace with changing social structures. Making divorce more socially acceptable, and reducing the stigmatization and victimization of divorcees may help prevent couples from staying in an unhappy marriage out of force.

This can be achieved through education and by having open conversations about interpersonal dynamics in spousal relationships, seeking professional help when problems occur, and not trivializing or ignoring the emotional repercussions of marital discord. Related information could also be included in a course on marriage counseling for university students or a mandatory premarital course offered through mental health professionals or social/religious institutions. The content of the course should focus on preparing couples for marriage by addressing many psychological and social disorders and problems such as alexithymia, such that emotional skills are developed clearly and realistically.

## Electronic supplementary material

Below is the link to the electronic supplementary material.

Supplementary Material 1

## Data Availability

The datasets generated and/or analysed during the current study are not publicly available due to ensure participants’ confdentiality but are available from the corresponding author on reasonable request.

## Abbreviations

TAS-20: Toronto Alexithymia Scale.
