# Supplementary material for: Relationship between emotional divorce and alexithymia among married women in Saudi Arabia
Source: BMC Psychol. 2023 Aug 2;11:217. doi: 10.1186/s40359-023-01236-w (PMC10398916; doi:10.1186/s40359-023-01236-w)
Supplement: Supplementary file 1 — Supplementary Material 1 [file 40359_2023_1236_MOESM1_ESM.pdf]

Princess Nourah Bint Abdulrahman University

Demographic Questions for Survey

1. Age: What is your age?
   a. 18-25
   b. 25-30
   c. 30-35
   d. 35 or older

2. Gender: What is your gender?
   a. Male
   b. Female

3. Can you read Arabic?
   a. Yes
   b. No

4. Employment: Are you an employee?
   a. Yes
   b. No

5. Children: How many children do you have?
   a. ≤1 children
   b. 2-4 children
   c. ≥ 5 children

6. Marital Status: What is your marital status?
   a. Single
   c. Married
   d. Separated
   e. Divorced
   f. Widowed

7. Marriage duration: How long is marriage?
   a. ≤ 4 years
   b. 5-9 years
   c. ≥ 10 years

8. Do you have an intellectual disability?
    a. Yes
    b. No

9. What’s your nationality?
    a. Saudi
    b. Non-Saudi
